# Three-dimensional totally endoscopic quadruple procedure in a high-risk patient who is a Jehovah's Witness: A comprehensive bloodless strategy

**DOI:** 10.1016/j.xjtc.2026.102506

**Published:** 2026-07-09

**Authors:** Joon Young Kim, Jae Suk Yoo

**Affiliations:** Department of Thoracic and Cardiovascular Surgery, Asan Medical Center, University of Ulsan College of Medicine, Seoul, Republic of Korea

**Figure:**
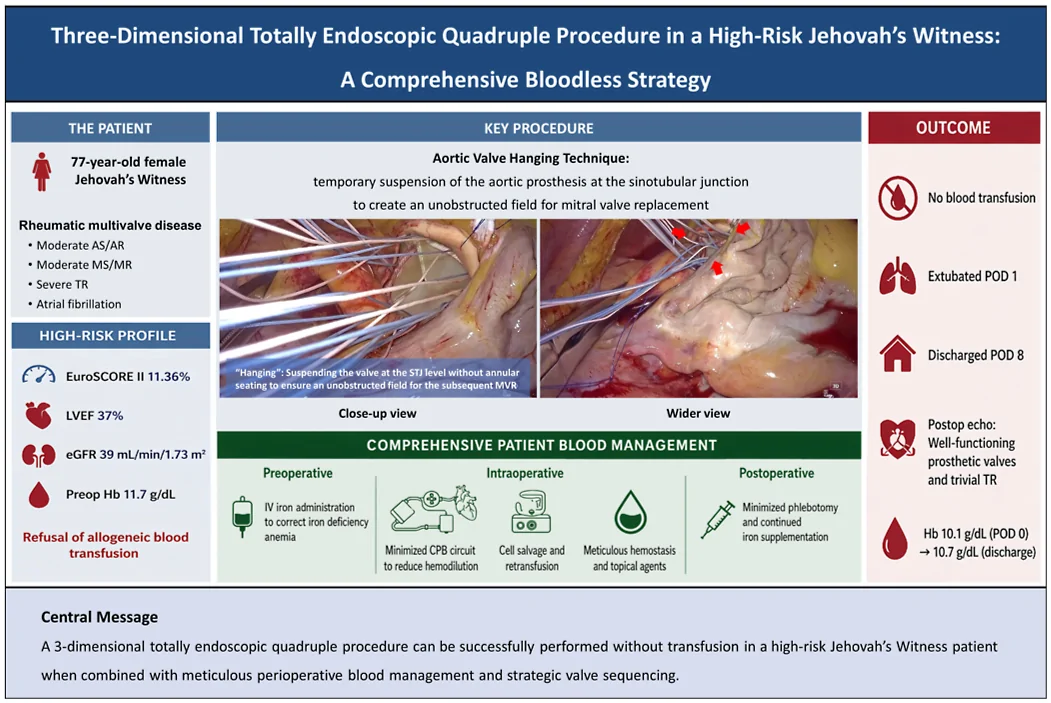
Bloodless 3D totally endoscopic quadruple procedure in a Jehovah's Witness patient.

**Central Message**

A 3D totally endoscopic quadruple procedure may be feasible without transfusion in a high-risk patient who was a Jehovah's Witness with meticulous blood management and strategic valve sequencing.

Cardiac surgery in patients who are Jehovah's Witnesses (JWs) presents a unique clinical challenge, given that allogeneic blood transfusion is not permitted. Perioperative patient blood management (PBM) strategies have been reported in this population,^1^ and minimally invasive concomitant aortic and mitral valve surgery has been described.^2^ However, experience with a 3-dimensional (3D) totally endoscopic quadruple procedure, defined in this report as aortic and mitral valve replacement, tricuspid annuloplasty, and surgical treatment of atrial fibrillation, including surgical ablation and left atrial appendage exclusion, remains limited. We report such a case using a comprehensive perioperative PBM strategy and a novel aortic valve (AV)-first sequencing technique with temporary aortic prosthesis hanging.

## Case Presentation

A 77-year-old woman who was a JW with a 4-year history of persistent atrial fibrillation presented with New York Heart Association class III dyspnea and rheumatic multivalve disease. Echocardiography showed moderate aortic stenosis/regurgitation, moderate mitral stenosis/regurgitation, and severe tricuspid regurgitation; preoperative imaging and clinical details are summarized in Video 1 and Table 1. Operative risk was high (European System for Cardiac Operative Risk Evaluation II, 11.36%), with reduced left ventricular ejection fraction (37%), renal dysfunction (estimated glomerular filtration rate, 39 mL/min/1.73 m^2^), and hemoglobin level of 11.7 g/dL. Given the rheumatic mixed stenotic-regurgitant pathology of both left-sided valves, double-valve replacement was planned. Bioprostheses were selected because of the patient's advanced age and the need to avoid lifelong warfarin therapy. This case report was approved by the institutional review board of Asan Medical Center (no. 2026-0497; April 10, 2026), which waived the requirement for informed consent.

**Table 1:** Preoperative, postoperative, and follow-up clinical findings

Variables	Preoperative	Postoperative	At discharge	3-mo follow-up
Anemia workup				
Ferritin, ng/mL	423.8	—	—	—
Serum iron, μg/dL	115.0	—	—	—
Transferrin, mg/dL	265.0	—	—	—
Total iron-binding capacity, μg/dL	327.0	—	—	—
Transferrin saturation, %	35.2	—	—	—
Red cell distribution width, %	12.9	—	—	—
Laboratory findings∗				
Hemoglobin, g/dL	11.7	10.1	10.7	Not yet available
Hematocrit, %	36.9	33.2	33.6	Not yet available
Platelet count, ×10^3^/μL	214	112	254	Not yet available
PT-INR	0.95	1.23	—	
aPTT, s	25.3	31.7	—	
eGFR, mL/min/1.73 m^2^	39	39	44	Not yet available
Echocardiographic/rhythm findings†				
LVEF, %	37	28	—	—
Valve findings	Rheumatic pathology with moderate AS/AR, moderate MS/MR, and significant TR	Well-functioning AV/MV prostheses, trivial residual TR		Not yet available
Estimated systolic PAP, mm Hg	52.0	38.0		Not yet available
RV function	Reduced; TAPSE 11.6 mm, RV S′ 5.0 cm/s	Reduced; TAPSE 6.0 mm,RV S′ 3.5 cm/s		Not yet available
LA volume index, mL/m^2^	104.1	96.5		Not yet available
Rhythm status	Atrial fibrillation	Atrial fibrillation	Normal sinus rhythm	Not yet available

*PT-INR*, Prothrombin time-international normalized ratio; *aPTT*, activated partial thromboplastin time; *eGFR*, estimated glomerular filtration rate; *LVEF*, left ventricular ejection fraction; *AS/AR*, aortic stenosis/aortic regurgitation; *MS/MR*, mitral stenosis/mitral regurgitation; *TR*, tricuspid regurgitation; *AV/MV*, aortic valve/mitral valve; *PAP*, pulmonary artery pressure; *RV*, right ventricular; *TAPSE*, tricuspid annular plane systolic excursion; *RV S*′, right ventricular peak systolic velocity; *LA*, left atrial.

∗ Postoperative laboratory values represent immediate postoperative findings.

† Postoperative echocardiographic findings were obtained on postoperative day 3.

## Surgical Technique

Peripheral cardiopulmonary bypass was established through femoral vessels and internal jugular vein. A 3D endoscopic system (Karl Storz), stabilized by a robotic scope holder (SOLOASSIST; AKTORmed), was used for visualization. All steps were performed endoscopically without direct vision through the working port.

A 3- to 4-cm working port was created in the third intercostal space at the right midclavicular line, and a 10-mm endoscopic trocar was inserted into the fourth intercostal space at the posterior axillary line. Myocardial protection was achieved using modified bloodless del Nido cardioplegia.^3^ After an oblique aortotomy, the AV was excised.

The first key technical step, AV-first annular stitching, was performed immediately after oblique aortotomy and AV excision. Figure-of-eight sutures were placed in the aortic annulus before any mitral work was to preserve the mitral-aortic intervalvular fibrosa (MAIVF) mobility and favorable needle angles in the restricted endoscopic field.

The second key step, the AV-hanging technique, temporarily suspended the aortic prosthesis (INSPIRIS RESILIA, 23 mm; Edwards Lifesciences) at the sinotubular junction, creating an unobstructed operative field for mitral valve replacement (Mosaic, 27 mm; Medtronic) and left-sided surgical ablation (Figure 1). The prosthesis was then seated under 3D endoscopic visualization, with annular seating confirmed during COR-KNOT (LSI Solutions) fastening. The procedure was completed with tricuspid annuloplasty (Tri-Ad, 26-mm ring; Medtronic), right-sided surgical ablation, and left atrial appendage exclusion with an AtriClip (AtriCure) via the transverse sinus. These key technical steps are demonstrated in Video 2.

**Figure 1:**
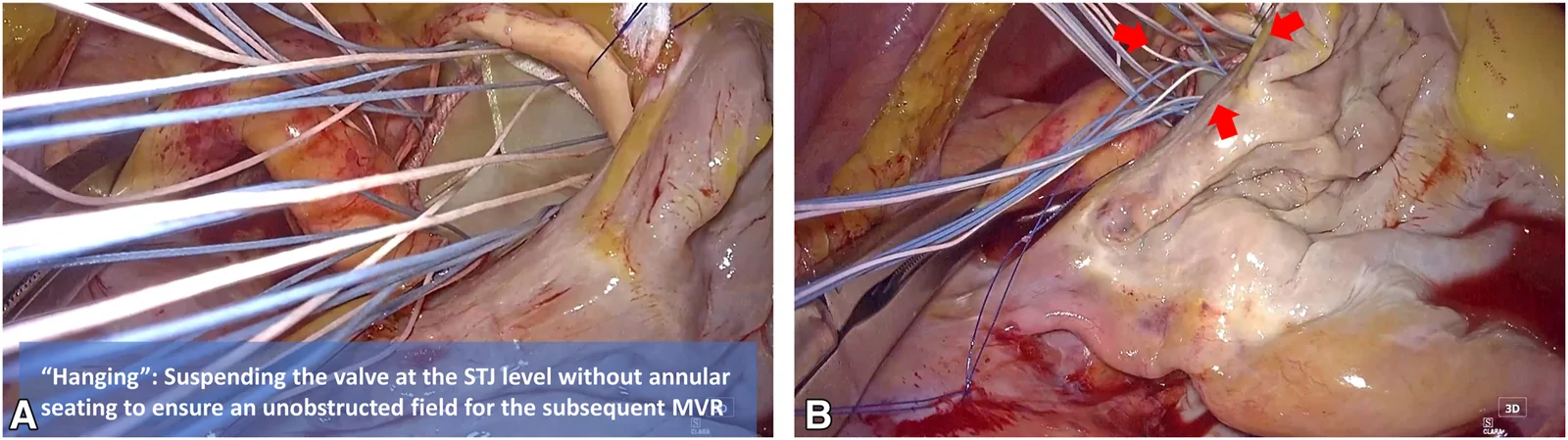
The aortic valve—hanging technique: temporary suspension of the aortic prosthesis at the sinotubular junction for mitral valve replacement. A, Close-up view of the suspended prosthesis without annular seating. B, Wider operative view showing the unobstructed field for left atriotomy and mitral valve replacement. *Red arrows* indicate an unobstructed operative field achieved by the AV-hanging technique.

## Postoperative Course

The patient was weaned from cardiopulmonary bypass without allogeneic transfusion. Intraoperative and postoperative variables are summarized in Table 2. No major adverse events occurred. The patient was extubated on postoperative day 1 and discharged on postoperative day 8. Postoperative echocardiography showed well-functioning prosthetic aortic and mitral valves and trivial residual tricuspid regurgitation (Video 3). A direct oral anticoagulant was administered for recurrent postoperative atrial fibrillation, with careful monitoring for bleeding. Hemoglobin was 10.1 g/dL immediately postoperatively and 10.7 g/dL at discharge.

**Table 2:** Intraoperative variables and postoperative outcomes

Variables	Value
Total operative time, min	331
Cardiopulmonary bypass time, min	201
Aortic crossclamp time, min	162
Estimated blood loss, mL	30–50
24-h chest tube drainage, mL	510
Drainage character	Serosanguineous
Aortic prosthesis	INSPIRIS RESILIA, 23 mm
Mitral prosthesis	Mosaic, 27 mm
Tricuspid annuloplasty ring	Tri-Ad, 26 mm
Mechanical ventilation time	15 h 45 min
ICU length of stay, d	2
Postoperative hospital stay, d	8
Allogeneic blood transfusion	No
Major adverse events∗	None

*ICU*, Intensive care unit.

∗ Low cardiac output syndrome, acute kidney injury, renal-replacement therapy, re-exploration for bleeding, stroke, infection, major bleeding event, and in-hospital mortality.

## Perioperative PBM

PBM was integrated across all phases.^4^ Routine preoperative iron studies (Table 1) showed no iron deficiency, and hemoglobin was 11.7 g/dL; therefore, neither ferric carboxymaltose nor erythropoietin was administered. At our institution, ferric carboxymaltose is reserved for patients with hemoglobin <10 g/dL approximately 1 month before surgery. The patient refused allogeneic red blood cells, fresh-frozen plasma, and platelets but accepted intraoperative cell salvage and residual cardiopulmonary bypass circuit blood retransfusion. Tranexamic acid was administered intraoperatively as a 10 mg/kg loading dose, followed by 2 mg/kg/h infusion. Meticulous hemostasis, topical hemostatic agents, and minimized postoperative phlebotomy were used.

## Discussion

This case supports the feasibility of a 3D totally endoscopic quadruple procedure without allogeneic transfusion in a high-risk patient who was a JW using multimodal PBM. Current PBM guidelines and previous data in patients who are JWs support meticulous hemostasis and minimally invasive access to reduce bleeding.^4^^,^^5^

The principal technical contribution is a 2-step sequencing strategy for totally endoscopic double-valve replacement. AV-first annular stitching preserved MAIVF mobility and favorable needle angles, and temporary AV hanging maintained exposure for mitral valve replacement and left-sided ablation before final prosthesis seating, reducing interference between procedural phases.

## Conclusions

This case demonstrates the feasibility of a 3D totally endoscopic quadruple procedure without allogeneic blood transfusion in a carefully selected high-risk patient who was a JW with comprehensive perioperative PBM. AV-first annular stitching followed by the AV-hanging technique may facilitate exposure, preserve MAIVF mobility, and improve technical efficiency during totally endoscopic double-valve replacement.

## Conflict of Interest Statements

The authors reported no conflicts of interest.

The *Journal* policy requires editors and reviewers to disclose conflicts of interest and to decline handling or reviewing manuscripts for which they may have a conflict of interest. The editors and reviewers of this article have no conflicts of interest.

## Supplementary Data

Video 1 Preoperative echocardiographic findings. Video available at: https://www.jtcvs.org/article/S2666-2507(26)00313-5/fulltext.

Video 2 Bloodless 3-dimensional totally endoscopic quadruple procedure in a high-risk patient who was a Jehovah's Witness. Video available at: https://www.jtcvs.org/article/S2666-2507(26)00313-5/fulltext.

Video 3 Postoperative echocardiographic findings. Video available at: https://www.jtcvs.org/article/S2666-2507(26)00313-5/fulltext.
